# Genetic Diversity Enhances Restoration Success by Augmenting Ecosystem Services

**DOI:** 10.1371/journal.pone.0038397

**Published:** 2012-06-25

**Authors:** Laura K. Reynolds, Karen J. McGlathery, Michelle Waycott

**Affiliations:** 1 Department of Environmental Sciences, University of Virginia, Charlottesville, Virginia, United States of America; 2 School of Earth and Environmental Sciences, University of Adelaide, Adelaide, Australia; Swansea University, United Kingdom

## Abstract

Disturbance and habitat destruction due to human activities is a pervasive problem in near-shore marine ecosystems, and restoration is often used to mitigate losses. A common metric used to evaluate the success of restoration is the return of ecosystem services. Previous research has shown that biodiversity, including genetic diversity, is positively associated with the provision of ecosystem services. We conducted a restoration experiment using sources, techniques, and sites similar to actual large-scale seagrass restoration projects and demonstrated that a small increase in genetic diversity enhanced ecosystem services (invertebrate habitat, increased primary productivity, and nutrient retention). In our experiment, plots with elevated genetic diversity had plants that survived longer, increased in density more quickly, and provided more ecosystem services (invertebrate habitat, increased primary productivity, and nutrient retention). We used the number of alleles per locus as a measure of genetic diversity, which, unlike clonal diversity used in earlier research, can be applied to any organism. Additionally, unlike previous studies where positive impacts of diversity occurred only after a large disturbance, this study assessed the importance of diversity in response to potential environmental stresses (high temperature, low light) along a water–depth gradient. We found a positive impact of diversity along the entire depth gradient. Taken together, these results suggest that ecosystem restoration will significantly benefit from obtaining sources (transplants or seeds) with high genetic diversity and from restoration techniques that can maintain that genetic diversity.

## Introduction

Ecological restoration is the process of augmenting the recovery of a degraded, damaged, or destroyed ecosystem [1]. A typical restoration goal is to create a stable functional ecosystem, which provides ecosystem services similar to less impacted reference systems. Ecosystem resistance and resilience (stability), and the provision of ecosystem services, such as primary and secondary production are often positively correlated with measures of biodiversity [2]–[4]. Given the positive benefits of biodiversity, it is often incorporated into and used as a measure of restoration success [5]. While biodiversity is often measured as species diversity, biodiversity is a hierarchical concept that can be measured at the scale of ecological guilds down to species, and even to variability within species [6]. In communities dominated by a single foundation species, such as temperate seagrass meadows, kelp forests, or cattail marshes, genetic diversity may be the most appropriate measure of biodiversity. The term genetic diversity is often broadly used to describe a number of measures, all of which may be important [7]. The number of unique individuals within populations is more appropriately called genotypic diversity or clonal diversity, whereas heterozygosity (measured within an individual) and allelic diversity (measured at the population level) are true measures of genetic diversity.

The positive impacts of genetic diversity have been documented in a variety of systems. For example, planting genetically diverse varieties of crops tends to produce greater yields as well as resistance to herbivory and disease [8], [9]. Genetic bottlenecks and inbreeding in small or endangered populations often result in decreased levels of heterozygosity and reduced fitness levels [10], [11]. Natural and manipulated marine plant assemblages have shown positive associations between clonal diversity and both density and some measures of ecosystem function (habitat and nutrient cycling) after large-scale disturbances [12]–[15].

*Zostera marina* (eelgrass) meadows are ideal model systems for studying the relationship between genetic diversity and ecosystem functioning. Eelgrass is a broadly distributed species in the Northern Hemisphere with coverage on both the east and west coasts of both the Atlantic and Pacific oceans [16]. The natural range of genetic diversity measured in this plant is high both within and between meadows, probably due to its adaptability to a wide range of environmental conditions and to its ability to reproduce both sexually and asexually [17], [18], [13]. Previous work has also shown that genetic diversity was positively correlated with plant density and with the density and diversity of organisms that use the seagrass as a habitat [11], [12].

Seagrass genetic variability, as for all clonal plants, can be measured as either genotypic or genetic diversity. Positive effects of genotypic diversity (number of clones) can occur when one or a few individuals are particularly well adapted to local conditions. Theoretically where genotypic diversity is high, it is more likely that one or more individuals will be well adapted. While genotypic or clonal diversity measures the number of unique genetically-defined individuals or clones per area, it does not capture the degree of genetic variation among individuals like genetic diversity does. Heterozygosity (combinations of alleles within individuals) is the most often used measure of genetic diversity and is a measure independent of the number of alleles per population. Allelic richness (among all individuals in populations) captures the total diversity present in populations, but is independent of the combinations of alleles. A community with a high genetic diversity and an abundance of genotypes (and thus phenotypes) is likely to have individuals that are occupying various niches. Complementarity is a mechanism by which genetic diversity increase population fitness, and occurs when individuals have a variety of phenotypes, which allows the population access to different pools of resources, and thus limits competition. Increasing the pool of genetic diversity among clonal genotypes also improves evolutionary potential and adaptive capacity under changing environmental conditions.

As genetic diversity is more difficult to manipulate than clonal diversity in natural systems, most studies that have contributed to our understanding of the relationship between genetics and ecosystem function have primarily used clonal diversity as their measure of genetic diversity. Manipulative experiments have shown that as a result of disturbance, clonal diversity–measured as number of unique genotypes–improved habitat quality [12], [13] and plant resistance to further disturbance [11], [12], [14]. There is some evidence that genetic diversity–measured as heterozygosity–is positively correlated with eelgrass fitness [19], [20]. However, when analyzed in the same system, it is not clear that genotypic and genetic diversity have the same influence on either plant fitness [21] or habitat quality [14].

Previous results demonstrating that clonal diversity enhances ecosystem resistance to disturbance have been used to direct seagrass restoration [22]; however, their applicability to large-scale restoration is debatable since experiments did not replicate restoration conditions. Typical stresses that seagrass restoration efforts face include unfavorable light conditions due to sediment resuspension and nutrient-over-enrichment [22], [23], and bioturbation [24], [25]. However, the documented positive effect of clonal diversity has been shown only during or after very large, albeit natural, disturbance events: for example, a grazing event that removed up to 75% of the biomass [11], a warming event that has a return time of 10,000 years [12], and the largest macroalgal bloom recorded at a site in a 4 year period [14]. These large disturbances do occur in nature but are not typical, and the role of genetic diversity in providing resistance to the more persistent and common stressors is not clear.

We conducted a realistic field-based restoration experiment using techniques, sources, and restoration sites currently being employed by ongoing large-scale restoration to demonstrate that a small increase in genetic diversity to a system with high baseline diversity can improve restoration success when measured by the provision of ecosystem services (habitat, productivity, and stability). This benefit of genetic diversity is evident both with and without specific stresses and disturbances. Because our experimental system has high levels of heterozygosity with little variability, we use allelic diversity as our measure of genetic diversity. The outcomes of this study will be broadly applicable to understanding ecosystems and their restoration, and it is novel in that few studies have demonstrated the effects of allelic diversity on ecosystem functioning experimentally in the field.

## Results

**Figure 1:**
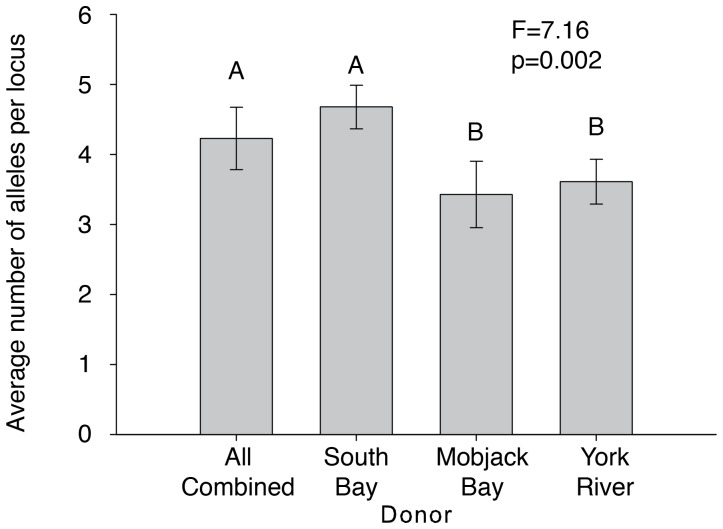
*Zostera marina* seeds were collected from Mobjack Bay and the York River in Chesapeake Bay and from South Bay, part of the Virginia coastal bay system. Seeds were planted in Hog Island Bay, also part of the Virginia coastal bay system in plots as either individual sources or as plots with all seed sources combined. Plots planted with seeds from either Mobjack Bay or the York River had a lower overall genetic diversity (measured by alleles per locus) than plots planted from either South Bay or from all three seed sources combined.

Plants in plots that were seeded from different sources differed in the average number of alleles per locus (F = 7.16, p = 0.002). Other experiments in this region have shown that regional genetic diversity of seagrass is high [26], and the plants in this experiment also had a high diversity relative to other studies. However, the experimental plots seeded both from South Bay seeds and all of the seeds combined had a greater number of alleles per locus (4.4±0.3 s.e.) than those plots seeded from the Chesapeake Bay sites of Mobjack Bay and the York River (3.5±0.3 s.e.) (Fig. 1).

**Figure 2:**
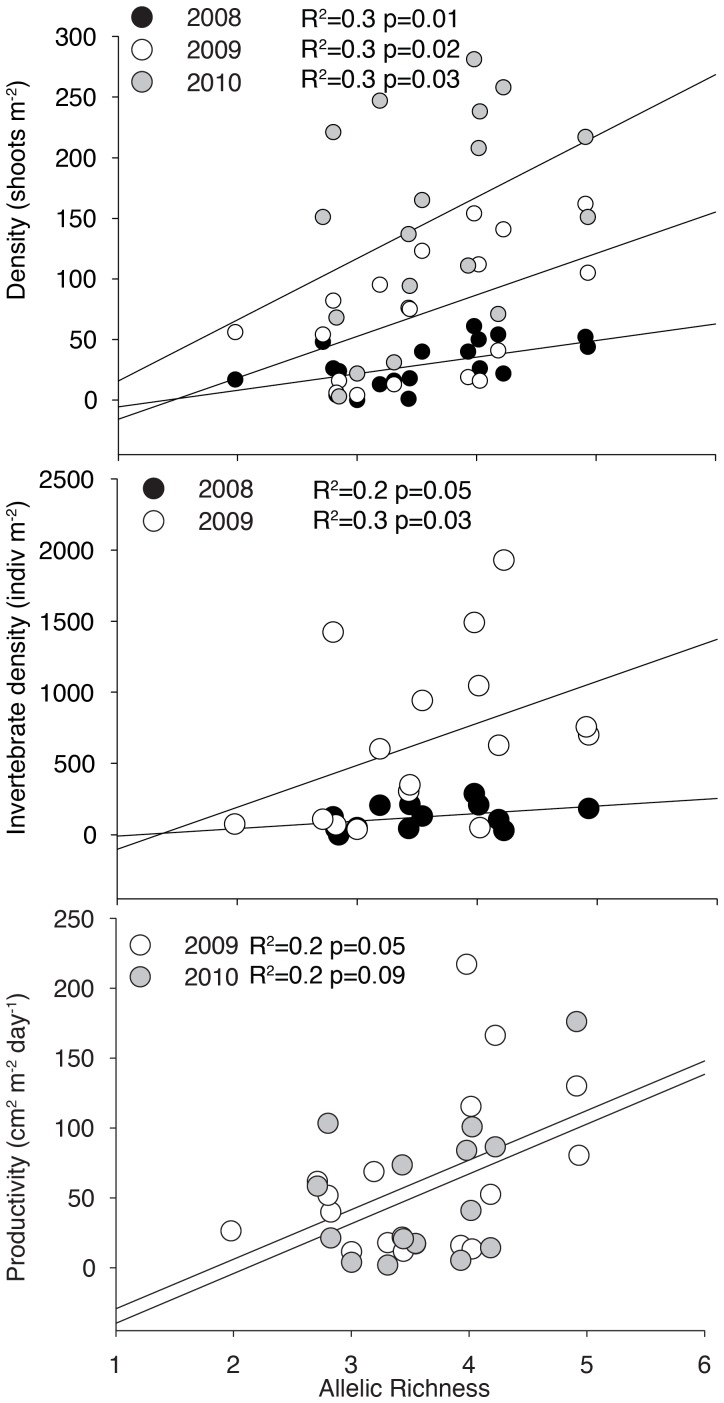
The relationship between plant density (A), invertebrate density (B), and areal productivity (C) during the peak growing season (June) was regressed against plot genetic diversity.

Measured genetic diversity was positively correlated with density and areal productivity during peak growing season (June) during the 3 years monitored (Fig. 2). The number of invertebrates was also positively correlated with genetic diversity during the summer (Fig. 2); however, in 2010, invertebrate density was measured during the fall and there was no significant relationship (R^2^ = 0.1, p = 0.5).

**Figure 3:**
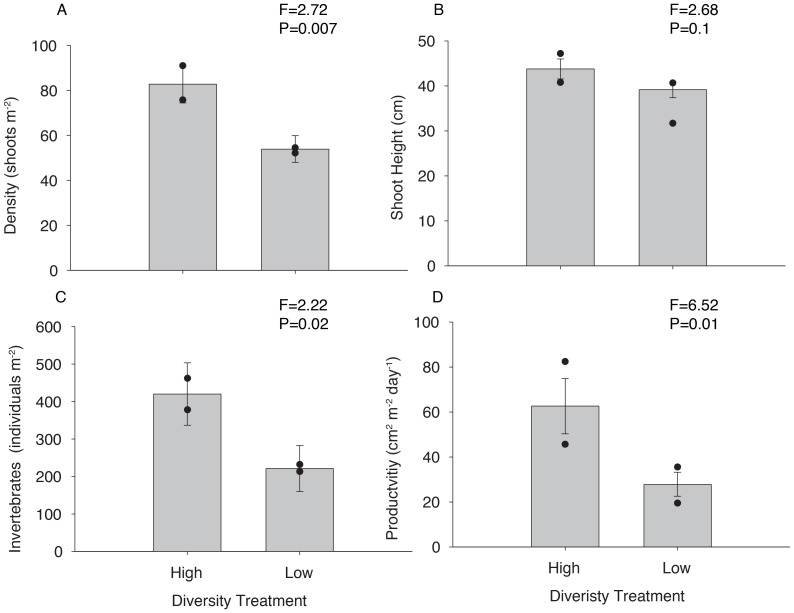
Experimental *Zostera marina* plots were planted in Hog Island Bay in two levels of genetic diversity: relatively high (4.4 alleles per locus ±0.3 s.e.) and relatively low (3.5 alleles per locus ±0.3 s.e.). During the peak summer growth (June and July), plant characteristics [density (A) and shoot height (B)] and measured ecosystem services [habitat function estimated as invertebrate density (C) and areal productivity (D)] were measured, and differences between high diversity and low diversity plots were analyzed with a t-test. Error bars represent standard error. Dots represent the mean of plots from individual seed sources.

We will refer to the South Bay and combined plots as ‘high diversity’ and the Mobjack Bay and York River plots as ‘low diversity’ even though both measures are high compared to other geographical regions [25] (Fig. 1). Although there was a difference in overall diversity, the two groups of populations were quite similar in genetic makeup, with a pairwise F_ST_ value of 0.01. During the months of high growth (June and July), plants in the high-diversity treatment were more dense (F = 2.72, p = 0.007) (Fig. 3a). Maximum height was marginally higher in high-diversity plots than in low-diversity plots (F = 2.68, p = 0.1) (Fig. 3b). Shoot-specific productivity did not differ between treatments (F = 0.4, p = 0.5), but because of increased density, overall areal productivity was higher in high-diversity plots (F = 6.52, p = 0.01) (Fig. 3c). Nitrogen content of the leaves did not vary with diversity treatment (F = 1.05, p = 0.5); however, because high diversity plots were more productive, they had higher nitrogen standing stock (F = 6.25, p = 0.01). Likewise, there was no difference in the number of invertebrates per shoot (F = 1.15, p = 0.6), but again since the high-diversity plots were more dense, there were more invertebrates per area in more diverse plots (F = 2.22, p = 0.02) (Fig. 3d).

**Figure 4:**
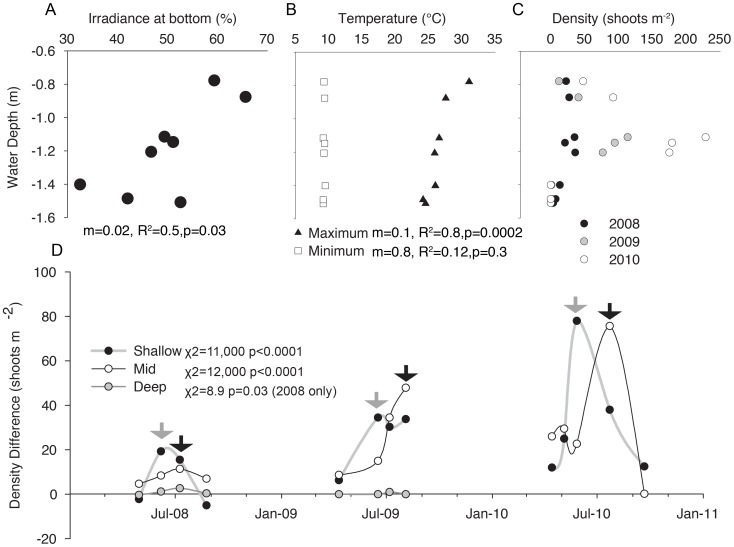
Experimental *Zostera marina* plots were planted in Hog Island Bay over a depth gradient of 0.8 m (range −0.78–1.5 MSL). Environmental conditions [light (A) and temperature (B)] varied with depth and resulted in differences in plant density (C). Plots at a depth less than 1 m and plots with a depth greater than 1.4 m had lower densities, while mid-depth plots had high densities that increased each year. Plots, replicated at each depth, were assigned to one of two levels of genetic diversity: relatively high (4.4 alleles per locus ±0.3 s.e.) and relatively low (3.5 alleles per locus ±0.3 s.e.). (D) Differences in density between the high diversity and low diversity plots were analyzed with a chi-square test (expected value of 0). Plants at the deepest depths died during the second growing season; therefore, differences were analyzed for the first year, while all plots had live plants, independently. Arrows indicate the timing of maximum density difference between high and low diversity plots.

Environmental characteristics varied over the depth gradient. Light decreased with depth (m = 0.02, R^2^ = 0.5, p = 0.03) (Fig. 4a), and while minimum daily temperature did not vary with depth (m = 0.8, R^2^ = 0.12, p = 0.3), maximum daily temperature was greater, often above 30°C, (m = 0.1, R^2^ = 0.8, p = 0.0002) in shallower water (Fig. 4b). Plant density and survival varied along the depth gradient. Shallow plots were less dense than plots at the moderate depth, and the density of plants at the moderate depth expanded throughout the three years (Fig. 4c). In both the shallow (temperature stressed) and mid-depth (relatively unstressed) blocks, high-diversity plots were more dense than low-diversity plots (Shallow: χ^2^ = 11,000, p<0.0001; Mid-depth: χ^2^ = 12,000, p<0.0001) (Fig. 4d). The plants in the deeper water died during the second growing season. During the first growing season, while all plots still had live plants (2008), the high-diversity plots at the deeper sites were consistently more dense than the low-diversity plots over all sampling months (χ^2^ = 8.9, p = 0.03). This positive relationship decreased during the second year as plants died and eventually the density of all of the deep plots became 0 (χ^2^ = 9.7, p = 0.2) (Fig. 4d). Because the plots were monitored monthly, an approximate time of survival (in months) could be determined. Plots with a higher genetic diversity lived about one month longer (F = 2.8, p = 0.1), suggesting that genetically diverse assemblages are more resistant to chronic light stress.

**Figure 5:**
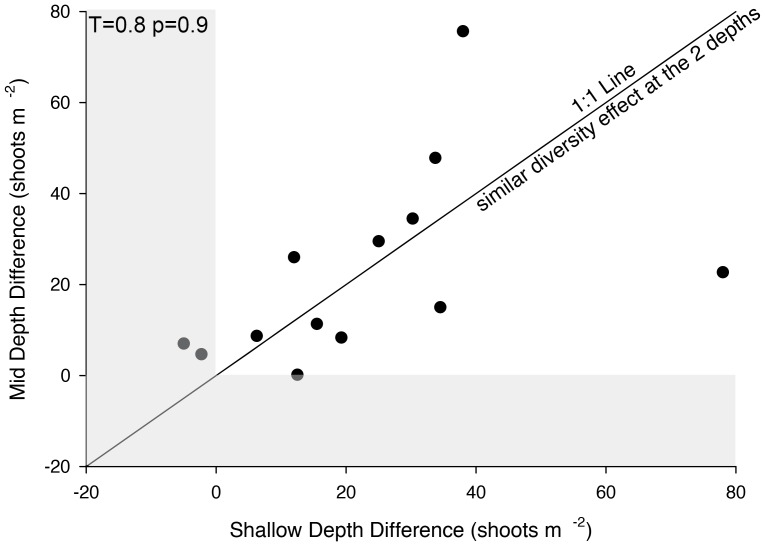
Experimental *Zostera marina* plots were planted in Hog Island Bay in two levels of genetic diversity: relatively high (4.4 alleles per locus ±0.3 s.e.) and relatively low (3.5 alleles per locus ±0.3 s.e.). Plots were replicated at depth. Plots at a depth less than 1 m were apparently heat stressed had reduced densities, while plots at depths between 1 and 1.4 m had high densities that increased over time. For each sampling date, a difference in density between high diversity and low diversity plots was calculated at each depth. A paired t-test (paired at sampling date) was used to determine if there was a greater effect of diversity on density at the different stress levels. The solid line is a 1∶1 line representing no differences in the effect of diversity between the two depths. The grey areas represent samples where the effect of genetic diversity on plant density was not significant.

High-diversity treatments were more dense across the environmental gradient; however, the magnitude of difference between high and low diversity treatment varied temporally. There was a consistent pattern where in June the difference between the high- and low-diversity plots was greatest when the plots were shallow and heat stressed, while in July the difference between the high- and low-diversity plots was greatest when plots were at a moderate depth and apparently unstressed (Fig. 4d). Despite temporal variability, the overall density difference between high- and low-diversity plots was consistent among shallow, heat-stressed plots and mid-depth, unstressed plots (Fig. 5).

## Discussion

Genetic diversity, measured as allelic diversity, was positively associated with seagrass density, which cascaded upward into positive impacts on invertebrate density, nitrogen retention, and areal productivity. This is in agreement with previous studies that have demonstrated a positive effect of clonal diversity on ecological parameters [11], [12], [14]; however, these results are unusual in that the enhancement of ecosystem services occurred without obvious signs of ecological stress or disturbance. The enhancement of ecosystem services also occurred despite a relatively small increase in genetic diversity in the region, which has been documented as one of the most diverse in the world [25]. These results suggest that the success of seagrass restoration will increase when efforts are made to use transplants with a high genetic diversity and with techniques that maintain that diversity (i.e. seeding; [25]).

Previous work has shown that genotypic diversity, measured as clonal richness, [11], [12], [14] and genetic diversity, measured as heterozygosity [19], [20], were positively associated with plant fitness and ecosystem stability. Our field experiment demonstrates a similar relationship with allelic diversity. This is significant since previous measures of diversity (clonal richness and heterozygosity) are not appropriate for many systems. Because our experimental restoration plots were initially planted with seed, all recruits were genetically distinct and as a result clonal diversity would be 1.0 at the start of the experiment for all sites. Because clonal diversity was constant, and we did not detect any clonal dominance across the experiment, it was an unsuitable measure to describe variability. In addition, heterozygosity was very high and largely invariant [25], making this also unsuitable for describing variability. Allelic diversity, however, was a robust measure of diversity and could also be applicable in most other systems. It should be noted that it is clear from the literature that genotypic diversity is potentially important for *Zostera marina* [12]–[15] and that over time it is possible that genotypic diversity may become a more important measure in this system once the effect of initial establishment is overcome.

Allelic diversity was associated with increases in both seagrass density and ecosystem services, including habitat provision, productivity, and nitrogen retention. The mechanism for this enhancement is the increase in density, as there was not a shoot-specific increase in ecosystem services. In this study, as well as another in the same system, density was an appropriate measure of restoration success and a suitable variable for understanding the impact of genetic diversity on seagrass ecosystems [27].

This study found a positive impact of allelic diversity on density under different environmental stress regimes: chronic light stress that killed the plants, temperature stress that decreased density, and low stress levels with no apparent effect on the plant. While the importance of diversity in terrestrial systems has been shown in the absence of stress [28], this is one of the first studies to show the positive effect of genetic diversity under low stress conditions in seagrass systems. The overall relationship between density and diversity did not differ in the temperature-stressed shallow water and the low-stress moderate depths (Fig. 5); however, in temperature-stressed plots, there was an earlier separation of high-diversity and low-diversity plots both initially and during each season sampled (Fig. 4d). This suggests that diversity is important regardless of stress, but disturbance or stress can cause a shift in response. We hypothesize that when stressed, plants require more resources, causing competition and earlier importance of niche complementarity than in systems where plants are not stressed and not severely resource limited. Plants in the deep plots, which experienced the lethal low-light stress, acted similarly to all other plants during the first year (Fig. 4d). During the second year, plants started to die and patterns were more difficult to detect, but more genetically diverse plots lived longer, showing some ecosystem resistance to the stress that led to their death.

This increased stability is similar to past results using clonal diversity. Manipulative experiments have shown that experimental plots with a larger number of individual clones (range 1–8) have better resisted large disturbances due to geese grazing [11], extreme temperature events [12], and large macroalgal blooms [14]. Our findings are important and unique since they were observed under a common chronic stress rather than a large disturbance. One of the most common causes of seagrass decline is decreased water quality, which promotes planktonic and epiphytic algal growth reducing light levels and shading seagrasses [29]. While plants in our experiment were not resilient and did die, if the reduction in light was shorter term such as occurs with sediment suspension during a storm event or a short-term nutrient pulse, the plants that survived longer may have outlived the disturbance and continued to survive.

Both complementarity and dominance of a few genotypes have been described as mechanisms for genotypic diversity enhancement of disturbance resistance in other studies [14]. For strong dominance to occur, a small number of genotypes would need to be more abundant. We found no clones in our analysis, and this is typical of this region, where flowering rates, seed production, and clonal diversity are high [25]. Further, overall low pairwise F_ST_ values suggest that there may not be many unique alleles in these more diverse populations. Instead there must be more combinations of similar alleles, which is more likely to lead to complementarity as opposed to dominance.

One of the strengths of our experiment is that it replicates realistic conditions for restoration. Seeds were collected from sites that are used regularly for restoration projects and planted in the same manner and in close proximity to a site that is being used for large-scale restoration [25], [26]. Our results suggest restorations that achieve high levels of genetic diversity will be more successful and will create more resilient seagrass ecosystems where plants survive longer, reproduce more rapidly, more quickly increase in density, and provide more ecosystem services.

## Materials and Methods

### Experimental Setup

In May of 2007, flowering shoots were collected from three sites: (1) Mobjack Bay (UTM 18S 384784 E 4127127 N); and (2) the York River in Chesapeake Bay (UTM 18S 374285 E 4125059 N), and (3) South Bay (UTM 18S 428005 E 4124724 N), which is part of the Virginia coastal bay system. Seeds from the flowers were then used in a restoration experiment at Hog Island Bay (UTM 18S 435429 E 4140648 N), also part of the Virginia coastal bay system. Hog Island Bay and South Bay are part of the Virginia Coast Reserve Long Term Ecological Research (LTER) site. All necessary permits were obtained for the described field studies. The restoration site is part an area set aside for seagrass research and restoration by the Virginia Marine Resources Commission and access and collection were permitted through collaboration with the Virginia Institute of Marine Sciences.

In Hog Island Bay 32, 2 m×2 m plots were seeded at a density of 100 seeds m^−2^ using an approach that has been successfully applied to restoration in this region [30], [31]. Seeds were distributed underwater and gently covered with sediment by hand. The plots were distributed in 8 blocks of 4 plots each along a depth gradient of about 0.8 m (range −0.78–1.5 MSL). To establish variation in genetic diversity in the experiment, in each block one plot was planted with seeds from each of the three source populations, and the fourth plot was planted with seeds from all of the source populations combined. The experiment was monitored over three growing seasons.

Twice during the experiment, differences in light and temperature conditions along the depth gradient were analyzed. Temperature was monitored for one month using HOBO temperature loggers that read every 15 min. Light profiles were taken at the center of each block using a LiCor spherical 4 Pi sensor (n = 3 for each block).

The genetic diversity of the plants in each plot was measured once during the experiment, 20 months after seeding. Six mature shoots from each plot were collected. At the time of collection, this was more than 10% of total shoots. DNA was extracted from each shoot using Qiagen DNeasy plant extraction kits, DNA was amplified at 8 microsatellite loci (loci: CT17H, CT3, CT35, GA2, CT19, CT20, GA3, and GA6) using standard PCR techniques [32], and fragments were analyzed by capillary electrophoresis on a MegaBACE 1000 (GE Biosciences) with an internal ET ROX 400 size standard (GE Biosciences).

Shoot density, invertebrate density, seagrass productivity, and leaf nitrogen content were measured in each of the plots for three growing seasons (2008–2010). Monthly density (May–August) was counted in four haphazardly placed 0.25 m^2^ quadrats, and the maximum height of five haphazardly chosen shoots were measured. Once during the growing season, invertebrate density and productivity were estimated. Three shoots from each plot were carefully extracted and preserved in isopropyl alcohol until invertebrates could be sieved, counted, and identified in the laboratory. Plant productivity was estimated by marking all shoots in a 0.01 m^2^ quadrat [33]. Three independent samples of young tissue were taken, dried, ground and analyzed on a Carlo Erba Elemental Analyzer.

### Data Analyses

We assessed differences in genetic diversity among the different seed sources. For every plot an average of all alleles at each of the 8 loci was calculated using GenAlEx 6.3 [34]. Differences in diversity between treatments were analyzed using ANOVA followed by Tukey pairwise comparisons. Data were log transformed to meet the assumption of homogeneity of variance. The overall difference in genetic makeup between groups was analyzed using F_ST_ (estimated as Q, [35]) in the software package Genodive v 2.0 [36].

Since the numbers of alleles per locus were statistically higher for the South Bay and all combined treatments than for the Mobjack Bay or York River donors, we refer to these combined treatments as relatively high (South Bay donor and all donors) and relatively low (York River and Mobjack Bay both in Chesapeake Bay). To determine overall differences in density and the provision of ecosystem services, summer data were pooled by block and by the diversity categories. Differences in pooled data were analyzed with a t-test. In addition, the direct relationship of measured parameters to genetic diversity was explored with a regression.

Differences in light and temperature conditions with depth were analyzed using a standard regression. The potential impact of differences in temperature and light to the plants was considered by comparing the mean density of all plots at each depth.

Data were also blocked into three groups by dividing the depth gradient into three equal parts. Environmental stresses varied among those depth groups: higher temperatures in shallow depths, which often rose above 30°C (<0.9 m), lower light in deeper water that led to eventual mortality as predicted from models [37] (>1.3 m), and less stressful conditions with respect to light and temperature at moderate depths (0.9–1.3 m). We analyzed the impacts of genetic diversity on seagrass density at each of these depth intervals. For each block, a difference between high-diversity treatments and low-diversity treatments was calculated, and a chi-square goodness of fit test with an expected value of 0 (no effect of genetic diversity) was conducted. Differences between the effect of genetic diversity on density under separate stress regimes (shallow temperature stressed and mid-depth unstressed) was analyzed using a paired t-test, pairing the difference between high- and low-diversity treatments at each sampling date.

For those plots that did not survive the light stress, the length of time that each of those deeper plots survived was plotted against depth. A blocked ANOVA (block: diversity treatment, factors: water depth and block) was used to determine if the plants in the high-diversity treatment were more resistant to the light stress of deep water and thus survived for a longer amount of time.
